# Elevated Alpha 1(I) to Alpha 2(I) Collagen Ratio in Dermal Fibroblasts Possibly Contributes to Fibrosis in Systemic Sclerosis

**DOI:** 10.3390/ijms23126811

**Published:** 2022-06-18

**Authors:** Soichiro Sawamura, Katsunari Makino, Maho Ide, Shuichi Shimada, Ikko Kajihara, Takamitsu Makino, Masatoshi Jinnin, Satoshi Fukushima

**Affiliations:** 1 Department of Dermatology and Plastic Surgery, Faculty of Life Sciences, Kumamoto University, 1-1-1 Honjo, Kumamoto 860-8556, Japan; sawamuraderma@gmail.com (S.S.); pu7cox@yahoo.co.jp (M.I.); shimashu_p@yahoo.co.jp (S.S.); kajiderma@gmail.com (I.K.); makinoderma@gmail.com (T.M.); s_fukushima@kumamoto-u.ac.jp (S.F.); 2 Department of Dermatology, Wakayama Medical University, Wakayama 641-0012, Japan; mjin@wakayama-med.ac.jp

**Keywords:** extracellular matrix, fibroblast, fibrosis, metabolism, systemic sclerosis, type I collagen

## Abstract

Systemic sclerosis (SSc) is characterized by excessive collagen deposition in the skin and internal organs. Activated fibroblasts are the key effector cells for the overproduction of type I collagen, which comprises the α1(I) and α2(I) chains encoded by *COL1A1* and *COL1A2*, respectively. In this study, we examined the expression patterns of α1(I) and α2(I) collagen in SSc fibroblasts, as well as their co-regulation with each other. The relative expression ratio of *COL1A1* to *COL1A2* in SSc fibroblasts was significantly higher than that in control fibroblasts. The same result was observed for type I collagen protein levels, indicating that α2(I) collagen is more elevated than α2(I) collagen. Inhibition or overexpression of α1(I) collagen in control fibroblasts affected the α2(I) collagen levels, suggesting that α1(I) collagen might act as an upstream regulator of α2(I) collagen. The local injection of *COL1A1* small interfering RNA in a bleomycin-induced SSc mouse model was found to attenuate skin fibrosis. Overall, our data indicate that α2(I) collagen is a potent regulator of type I collagen in SSc; further investigations of the overall regulatory mechanisms of type I collagen may help understand the aberrant collagen metabolism in SSc.

## 1. Introduction

Systemic sclerosis (SSc) is a multisystem autoimmune disease characterized by excessive extracellular matrix (ECM) protein deposition in the skin and internal organs [1]. The pathogenesis of fibrosis in SSc includes inflammation, aberrant immune activation, and endothelial cell injury, resulting in fibroblast activation to increase ECM production, mainly type I collagen [2,3]. Transforming growth factor-β (TGF-β) is one of the major profibrotic cytokines and the most important factor involved in fibroblast activation [4,5]. TGF-β is also known to play an important role in excessive ECM production in SSc [6]. However, the mechanisms underlying excessive type I collagen production have not been fully elucidated.

Type I collagen, the main product of abnormal collagen metabolism in SSc [3], is typically a heterotrimeric protein comprising two α1(I) collagen (encoded by *COL1A1* gene) polypeptides and one α2(I) collagen (encoded by *COL1A2* gene) polypeptide [7,8]. Although *COL1A1* and *COL1A2* gene expression is thought to be regulated in concert, their regulatory factors are complex and remain unclear [9]. Electron microscopy studies have shown that immature collagen fibrils with uniform diameters are observed in the lower dermis of SSc [10]. The irregularity of collagen fibers in SSc suggests an abnormality in collagen amounts as well as in the properties of collagen [10,11,12].

The excessive accumulation of type I collagen in SSc is well known, but little is known regarding the ratio of α1(I) and α2(I) collagen and how they regulate each other in this disease. The blockade of type I collagen-related signaling, mainly TGF-β, has received attention as an anti-fibrosis treatment in SSc [13,14], but there has been little research focused on the direct inhibition of α1(I) and α2(I) collagen. Therefore, in this study, we evaluated the possibility that α1(I) and α2(I) collagen levels are altered in SSc fibroblasts and examined whether the direct inhibition of type I collagen expression using small interfering RNA (siRNA) has anti-fibrotic effects in a bleomycin-induced SSc mouse model.

## 2. Results

### 2.1. The Ratio of α1(I) and α2(I) Collagen Is Increased in SSc Dermal Fibroblasts

**Figure 1:**
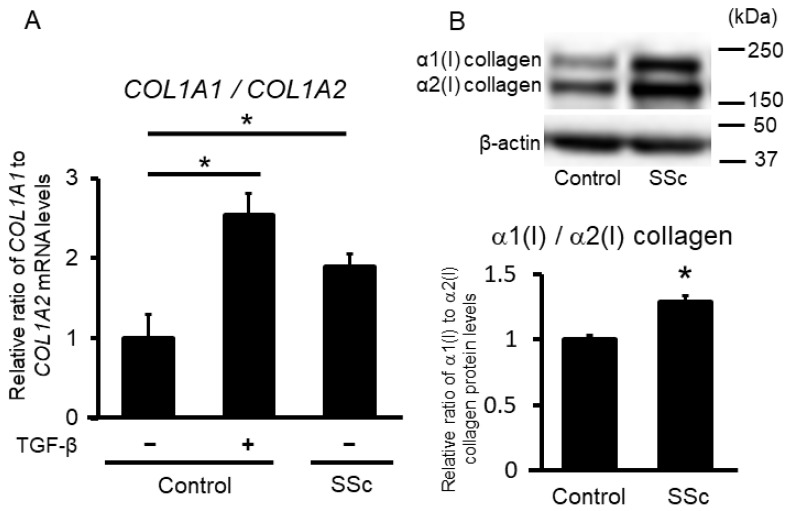
Relative ratio of type 1 procollagen in control and systemic sclerosis (SSc) dermal fibroblasts. (**A**) Relative ratios of *COL1A1* to *COL1A2* mRNA were determined using real-time PCR in control, and SSc fibroblasts stimulated with or without TGF-β (n = 3 per group). (**B**) Control and SSc fibroblast lysates were subjected to immunoblotting. The graph shows the relative ratio of the type I collagen α1 chain to the α2 chain between control and SSc fibroblasts using densitometry (n = 4 per group). Each graph presents the mean ± standard deviation. The mean value in the control group was set as 1. * *p* < 0.05.

In an initial experiment to evaluate the expression patterns of type I procollagen, we extracted total RNA and protein from SSc and control dermal fibroblasts, with and without TGF-β stimulation. The relative ratio of *COL1A1* to *COL1A2* mRNA in SSc fibroblasts and control fibroblasts with TGF-β stimulation was significantly higher than that in control fibroblasts without TGF-β stimulation (Figure 1A). The relative ratio of α1(I) to α2(I) collagen protein expression determined by immunoblotting also showed the same tendency (Figure 1B). These results indicate that α1(1) collagen is predominantly expressed in SSc dermal fibroblasts, and this imbalance in type I procollagen expression may be attributed to stimulation of SSc fibroblasts with intrinsic TGF-β, as described in the introduction.

### 2.2. Forced Expression of α1(I) Collagen Affects α2(I) Collagen Expression in Control Dermal Fibroblasts

**Figure 2:**
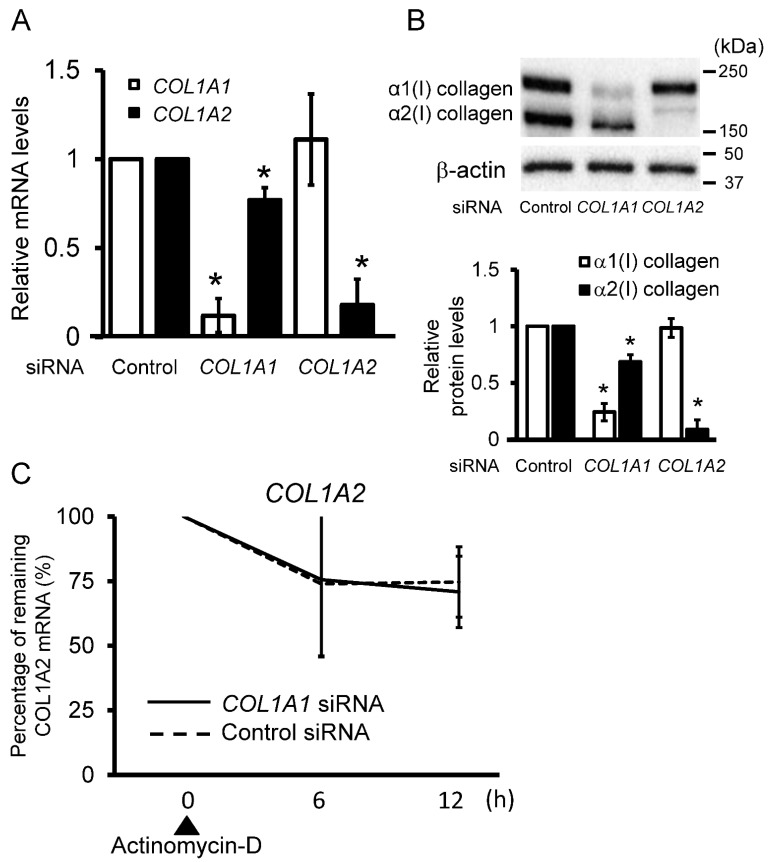
*COL1A1* silencing affects *COL1A2* transcript levels in fibroblasts. (**A**) Relative mRNA levels of type 1 procollagen in control fibroblasts transfected with *COL1A1* or *COL1A2* siRNA were determined using real-time PCR. The mean values in the control siRNA treatment were set as 1 (n = 3 per group). * p < 0.05 versus the control. (**B**) Protein levels of type I collagen in control fibroblasts transfected with *COL1A1* or *COL1A2* siRNA are shown. The graph shows the mean relative type I collagen levels. The mean value in control siRNA was set as 1 (n = 3 per group). * p < 0.05 versus the control. (**C**) Control fibroblasts transfected with *COL1A1* or control siRNA were incubated for 12 h after treatment with 2.5 μg/mL actinomycin D. *COL1A2* mRNA expression was analyzed using real-time PCR (and normalized to GAPDH). The values in untreated fibroblasts were set as 100% (n = 3 per group). * *p* < 0.05.

Little evidence is available regarding whether α1(I) and α2(I) collagen affect the expression of each other. Therefore, we performed experiments using control fibroblasts transfected with siRNAs targeting *COL1A1* and *COL1A2* expression. The depletion of *COL1A1* in the control fibroblasts reduced the expression of *COL1A2*, whereas silencing *COL1A2* did not affect *COL1A1* gene expression (Figure 2A). A similar trend was observed at the protein level (Figure 2B). To determine whether the down-regulation of *COL1A2* by *COL1A1* silencing occurred at the transcriptional level, we performed a stability assay of *COL1A2* mRNA using *COL1A1*-silenced control fibroblasts with actinomycin D stimulation. The relative decrease in *COL1A2* levels upon incubation with actinomycin D over time was not altered in the presence or absence of *COL1A1* siRNA (Figure 2C). This result indicated that *COL1A2* expression was decreased by *COL1A1* silencing without changing the stability of *COL1A2* mRNA. Therefore, we hypothesized that the α1(I) collagen level might act as an upstream regulator of α2(I) collagen expression.

**Figure 3:**
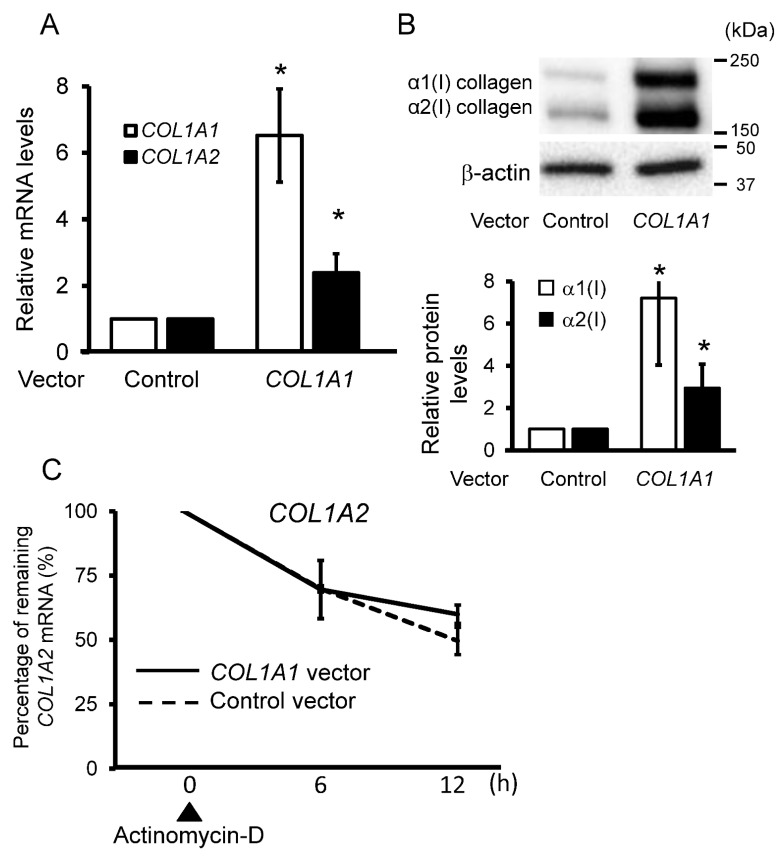
*COL1A1* overexpression affects *COL1A2* transcript levels in fibroblasts. (**A**) Relative mRNA levels of type 1 procollagen in control fibroblasts treated with virus-containing medium with a control or *COL1A1* expression vector were determined using real-time PCR. The mean value of the control vector was set as 1 (n = 3 per group). * p < 0.05 versus the control (**B**) Protein levels of type I collagen in control fibroblasts treated with virus-containing medium with a control or *COL1A1* expression vector are shown. The graph shows the mean relative type I collagen levels. The mean value in the control vector was set as 1 (n = 3 per group). * p < 0.05 versus the control. (**C**) Control fibroblasts transfected with virus-containing medium with a control or *COL1A1* expression vector were incubated for 12 h after treatment with 2.5 μg/mL actinomycin D. *COL1A2* mRNA expression was analyzed using real-time PCR (and normalized to GAPDH). The values in untreated fibroblasts were set as 100% (n = 3 per group). * *p* < 0.05.

To further test our hypothesis, we overexpressed *COL1A1* by lentiviral transfection in the control fibroblasts. As expected, the overexpression of *COL1A1* induced *COL1A2* mRNA expression (Figure 3A). Figure 3B shows the same tendency in protein expression levels. As shown in Figure 2C, we compared the stability of *COL1A2* mRNA between *COL1A1*-overexpressing and control fibroblasts. However, there was no significant difference in the stability of *COL1A2* mRNA between the two groups (Figure 3C). Based on these results, we hypothesized that *COL1A1* expression influences the transcriptional levels of *COL1A2*.

### 2.3. Local Administration of COL1A1 siRNA Attenuates Skin Fibrosis in a Bleomycin-Induced SSc Mouse Model

**Figure 4:**
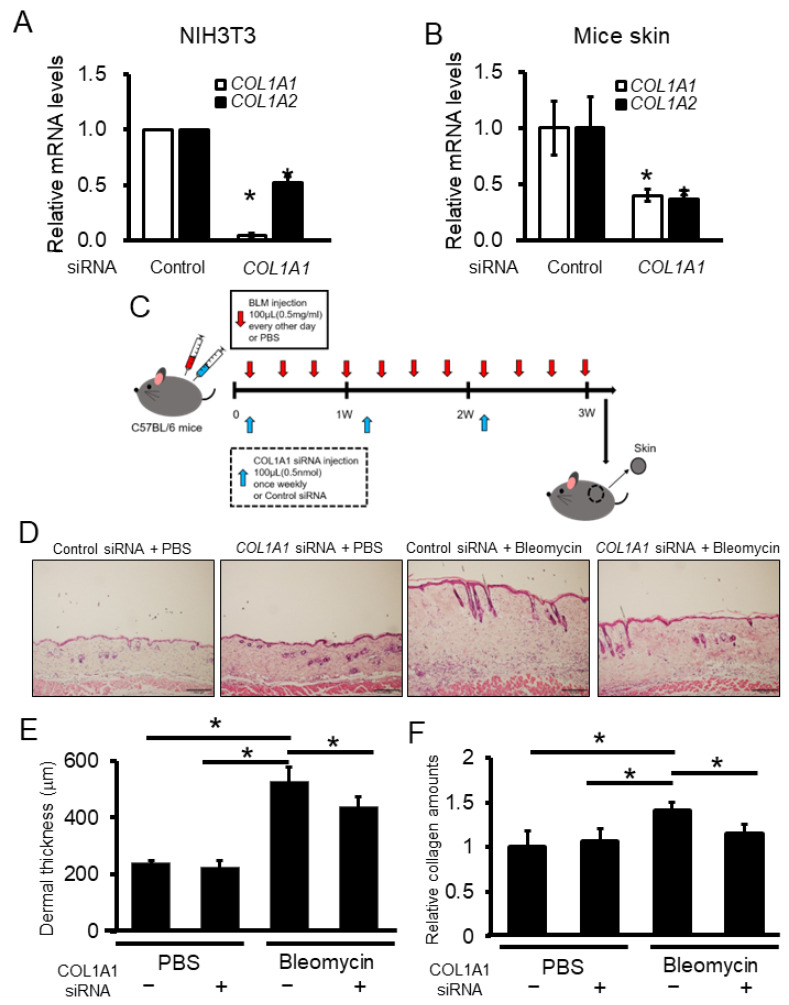
*COL1A1* silencing ameliorates skin fibrosis in a bleomycin-induced SSc mouse model. (**A**) Relative mRNA levels of type 1 procollagen in NIH3T3 cells transfected with *COL1A1* or *COL1A2* siRNA were determined using real-time PCR. The mean values of the control siRNA treatment were set as 1 (n = 3 per group). * *p* < 0.05 versus the control. (**B**) Relative mRNA levels of procollagen in mice skin injected with *COL1A1* or control siRNA were determined using real-time PCR. The mean values in the control siRNA treatment were set as 1 (n = 5 per group). * *p* < 0.05 versus the control. (**C**) The protocol for (**D**) to (**F**) is shown. Bleomycin or PBS were injected intradermally into the back skin of C57BL/6 mice every other day for three weeks. *COL1A1* or control siRNA mixed with atelocollagen were also injected into the back skin once weekly (for a total of three times). The back skin was obtained on the day after final bleomycin injection (**D**). Mouse skin sections were stained with hematoxylin and eosin. Representative results are shown. Scale bar = 200 μm. (**E**) Graph showing the results of dermal thickness (n = 5 per group) (**F**) Collagen content in mouse skin was measured using a hydroxyproline assay. Values are normalized relative to the PBS control group (n = 5 per group). * *p* < 0.05.

Considering the importance of α1(I) collagen, we examined whether the suppression of α1(I) collagen alone improves skin fibrosis in vivo. Prior to the in vivo experiments, we validated the effect of *COL1A1* siRNA using mouse fibroblasts (NIH3T3 cells). The mRNA expression of both *COL1A1* and *COL1A2* was significantly knocked down in *COL1A1*-silenced NIH3T3 cells (Figure 4A), indicating a similar tendency as in human control fibroblasts transfected with *COL1A1* siRNA. Furthermore, administration of *COL1A1* siRNA decreased the mRNA expression of both *COL1A1* and *COL1A2* in mouse skin compared with that of the control siRNA administration (Figure 4B).

Finally, we evaluated the anti-fibrotic effects of *COL1A1* siRNA in a bleomycin-induced SSc mouse model. Skin fibrosis induced using bleomycin injection in mice is a well-known model of SSc. Bleomycin was locally injected into the backs of C57BL/6 mice every other day for 3 weeks. Simultaneously, the control siRNA or *COL1A1* siRNA mixed with atelocollagen was injected into the back skin once weekly (Figure 4C). Bleomycin-induced mouse skin showed dermal fibrosis, with an increased number of thickened collagen bundles (Figure 4D). *COL1A1* siRNA significantly decreased dermal thickness and collagen content in the back skin of bleomycin-treated mice (Figure 4E,F). Collectively, these data indicate that *COL1A1* inhibition attenuates skin fibrosis in an in vivo model of SSc.

## 3. Discussion

Fibrosis is a key feature of SSc, resulting in fibroblast activation and ECM accumulation, especially type I collagen, which comprises two α1(I) and one α2(I) collagen chains. Type I collagen is usually produced at a 2:1 ratio of α1(I) and α2(I) collagen. We first demonstrated that the relative ratio of α1(I) to α2(I) collagen was higher in SSc fibroblasts than that in control fibroblasts, suggesting that α1(I) collagen is predominantly expressed in SSc fibroblasts. Previous studies have also reported that the ratio of α1(I) to α2(I) collagen is high in SSc fibroblasts [15,16,17,18]. The same tendency was observed in control fibroblasts stimulated with TGF-β. These results suggest that the biased increase in α1(I) collagen compared with that in α2(I) collagen may result from activated endogenous TGF-β signaling. TGF-β also acts as a pro-fibrotic cytokine in chronic graft-versus-host disease (GVHD), an autoimmune disease characterized by inflammation and fibrosis of the dermis and subcutaneous tissue. The aberrant expression of α1 and α2 collagens has also been reported in other diseases. The imbalance of α1(I) and α2(I) collagen owing to the lack of *COL1A2* has been reported in some orthopedic disorders and carcinomas [19,20,21]. Further, the loss of *COL1A2* results in the homotrimers of three α1(I) collagen polypeptides and is reported to induce alteration of collagen structure [22], strength [23], and molecular stability owing to collagenase resistance [24,25]. The details of this mechanism have not been elucidated in this study, and future studies are needed to clarify this point.

Although the *COL1A1* and *COL1A2* genes are located on separate chromosomes, their expression is regulated coordinately [9]. In this study, *COL1A2* expression was found to be altered with forced changes in *COL1A1* expression, suggesting that *COL1A1* expression acts as an upstream regulator of *COL1A2*. Additionally, an actinomycin D assay suggested that *COL1A1* influences the transcriptional level of *COL1A2* gene expression. We hypothesized that this could involve TGF-β because the biased expression of α1(I) collagen was found in the control fibroblasts upon TGF-β stimulation. Moreover, Dzobo et al. [26] reported that ECM components regulate the feedback pathway of *COL1A2* gene expression. Alterations in α1(I) collagen expression may affect the regulation of collagen synthesis via a similar pathway. Further, the ratio of *COL1A1* to *COL1A2* has been reported to be altered by microRNA-29 [27]; thus, post-transcriptional mechanisms could also be involved in this regulation. Further studies are needed to clarify these points.

Finally, we tried to determine the effect of administering *COL1A1* siRNA on skin fibrosis in a mouse model of SSc. siRNA technology has been widely studied for treating various diseases [28,29,30] and has attracted attention as a new approach for gene therapy in SSc [14,16,31]. Although previous reports have indicated the therapeutic effect of siRNA by knocking down the TGF-β signaling pathway, to the best of our knowledge, this is the first report describing the anti-fibrotic effect of *COL1A1* siRNA by directly knocking down the expression of type I collagen in mouse skin. The anti-fibrotic effect of *COL1A1* siRNA is assumed to have a direct as well as indirect inhibitory effect on type I collagen gene expression. For example, Vollmann et al. [32] indicated that *COL1A1* siRNA significantly reduced PDGFRβ mRNA levels in a mouse model of liver fibrosis because of the feedback loop between ECM accumulation and PDGFRβ. Our data also suggest a possible therapeutic application of *COL1A1* siRNA in patients with SSc; however, we could not elucidate its detailed anti-fibrotic mechanisms in this study.

In summary, this is the first report indicating the existence of a biased increase in α1(I) collagen compared with that in α2(I) collagen in SSc fibroblasts. There are only limited treatment options for patients with SSc. We demonstrated the potential of α1(I) collagen expression to affect α2(I) collagen levels. Therefore, inhibiting α1(I) collagen expression could be an efficient therapeutic strategy in SSc fibrosis. Although collagen metabolism in SSc remains unclear, further investigations regarding the regulation of type I collagen may facilitate a better understanding of SSc pathogenesis and provide new therapeutic approaches for this disease.

## 4. Materials and Methods

### 4.1. Reagents

Antibodies against type I collagen (1:1000, Cat.#1310-01) and β-actin (1:1000, Cat.#sc-47778) were purchased from Southern Biotechnologies (Birmingham, AL, USA) and Santa Cruz Biotechnology (Dallas, TX, USA), respectively. Recombinant human TGF-β1 (2 ng/mL, Cat.#240-B-002) was obtained from R&D Systems (Minneapolis, MN, USA). Actinomycin D (Cat.#018-21264) was purchased from Wako (Osaka, Japan).

### 4.2. Cell Cultures

SSc fibroblasts were obtained by skin biopsies from the affected areas (dorsal forearm) of three patients with diffuse cutaneous SSc and <2 years of skin thickening, as described previously [33]. Control fibroblasts obtained by skin biopsies from three healthy donors were purchased from the American Type Culture Collection (ATCC, Manassas, VA, USA) and Lonza (Walkersville, MD, USA). Mouse NIH3T3 cells were obtained from ATCC. Monolayer cultures of fibroblasts were maintained at 37 °C with 5% CO_2_. Cells were serum-starved for 12–24 h before all experiments [34]. All biopsies were performed according to the Declaration of Helsinki, with approval from the institutional review board, and written informed consent was obtained.

### 4.3. RNA Extraction, Reverse Transcription, and PCR Analysis of RNA Expression

The total RNA was extracted from cultured cells using ISOGEN (Nippon Gene, Tokyo, Japan). First-strand cDNA was synthesized using a PrimeScript^TM^ RT reagent kit (Takara Bio, Shiga, Japan). For quantitative real-time analysis, cDNA and primers were mixed with SYBR Premix Ex Taq^TM^ II (Takara Bio, Shiga, Japan). Primer sets for glyceraldehyde-3-phosphate dehydrogenase (GAPDH) and 18S ribosomal RNA (18SrRNA) were purchased from QIAGEN (Valencia, CA, USA) and Thermo Fisher Scientific (Waltham, MA, USA), respectively. Primer sets for human and mouse α1(I) and α2(I) collagen were obtained from Takara. The DNA was amplified over 40 cycles of denaturation for 5 s at 95 °C and annealing for 30 s at 60 °C, and relative expression was calculated using the ΔΔCt method [35].

### 4.4. Immunoblotting

The cultured human or mouse dermal fibroblasts were washed with PBS and lysed in RIPA buffer (Nacalai Tesque, Kyoto, Japan). Protein concentrations were quantified using a BCA Protein Assay kit (Thermo Fisher Scientific). Aliquots of cell lysates were separated by electrophoresis on 10% SDS-PAGE and transferred to polyvinylidene difluoride membranes, which were blocked in blocking One P buffer (Nacalai Tesque) for 1 h and incubated overnight at 4 °C with primary antibody. The membranes were washed with TBS and 0.1% TBST, probed with HRP-conjugated secondary Ab for 1 h, and then washed with TBST again [36]. Immunoreactive bands were visualized using the ChemiDoc XRS system (Bio-Rad, Hercules, CA, USA).

### 4.5. Transient Transfection

Human *COL1A1* siRNA (ON-TARGETplus SMART pool), *COL1A2* siRNA (ON-TARGETplus SMART pool), and control siRNA (ON-TARGETplus nontargeting control pool) were obtained from Dharmacon (Lafayette, CO, USA). Mouse *COL1A1* siRNAs (5′-UGGCCUUGGAGGAAACUUU-3′ and 5′-AAAGUUUCCUCCAAGGCCA-3′) and control siRNA were purchased from Nippon Gene (Tokyo, Japan). For transfection, siRNAs (20 nM) mixed with Lipofectamine RNAiMAX (Thermo Fisher Scientific) were added to cells in 24-well culture dishes, followed by incubation for 24–72 h at 37 °C in 5% CO_2_ [36].

### 4.6. Lentiviral Gene Transfer

Lentiviral vector-mediated gene transfer was performed using CSII-EF-RfA, pCMV-VSV-G-RSV-Rev, and pHIVgp, which were kindly donated by Dr. Hiroyuki Miyoshi (RIKEN, Wako, Japan) [37,38]. cDNA fragments of the full-length human *COL1A1* gene were generated and amplified by PCR using SuperScript^TM^ II Reverse Transcriptase (Invitrogen, Carlsbad, CA, USA) and PrimeSTAR^®^ HS DNA Polymerase with GC Buffer (Takara), followed by cloning into CSII-EF-RfA [39]. Substitution mutations were generated using the QuikChange Lightning Site-Directed Mutagenesis Kit (Agilent Technologies, Santa Clara, CA, USA) and were confirmed by sequencing [40].

### 4.7. Mice

To deliver mouse *COL1A1* siRNA into mouse skin, mixtures of siRNA and AteloGene^®^ Local Use (Koken, Tokyo, Japan) were prepared according to the manufacturer’s instructions. The *COL1A1* siRNA oligo was obtained from Koken:5′-UGGCCUUGGAGGAAACUUU-3′ and 5′- AAAGUUUCCUCCAAGGCCA-3′. An irrelevant control siRNA was also obtained from Koken:5′-AUCCGCGCGAUAGUACGUA-3′ and 5′-UACGUACUAUCGCGCGGAU-3′. The samples were then annealed with one another. Then, 100 μL of the mixtures (siRNA concentration, 5 μM) were administered into the dermis of six-week-old male C57BL/6 mice (CLEA Japan, Tokyo, Japan) once weekly [41]. All of the mouse experiments were performed in accordance with the guidelines of the Institutional Animal Committee of Kumamoto University and approved by the Committee on Animal Research at Kumamoto University.

### 4.8. Bleomycin Treatment in Mice

Bleomycin (Nippon Kayaku, Tokyo, Japan) was dissolved in PBS at a concentration of 0.5 mg/mL and sterilized by filtration as described previously [42,43]. Bleomycin (100 μL) was then injected intradermally into the shaved backs of 6-week-old male C57BL/6 mice (CLEA Japan, Tokyo, Japan) every alternate day for 3 weeks. The back skin was removed on the day after the final bleomycin injection, and fibrosis was evaluated by histological analysis and a total collagen assay. The control mice were injected with equal volumes of PBS. The dermal thickness was evaluated by measuring the distance between the epidermal-dermal junction and dermal-fat junction in hematoxylin-eosin sections.

### 4.9. Measurement of Collagen Production in Mouse Skin

The collagen deposition in 8-mm mouse skin punch biopsy samples was measured using a Total Collagen Assay kit (QuickZyme Biosciences, Leiden, Netherlands) following the manufacturer’s protocol. Briefly, mouse skin samples were hydrolyzed using 12 mol/L of hydrochloric acid for 20 h at 95 °C. The samples were then added to the microplate wells, and dilution assay buffer was added to each well. After incubation for 20 min at room temperature, the detection reagent was added to each well. The samples were incubated for 60 min at 60 °C, and the absorbance of each sample was read at 570 nm using a spectrophotometer. The results are expressed as the relative hydroxyproline content [44].

### 4.10. Statistical Analysis

The values are presented as the mean ± standard deviation. Statistical analyses were performed using the Mann–Whitney U-test for a comparison of the two groups. One-way analysis of variance (ANOVA) with Tukey’s post-hoc test was used for multiple comparisons. All of the analyses were performed using Statcel4 software (OMS, Kurume, Japan). The statistical significance was defined as *p* < 0.05.

## Data Availability

The datasets supporting the conclusions of this article are included in this published article.

## Abbreviations

ECM	extracellular matrix
siRNA	small interfering RNA
SSc	systemic sclerosis
TGF-β	transforming growth factor-β
